# Disease burden in a large cohort of asylum seekers and refugees in Germany

**DOI:** 10.7189/jogh.11.04002

**Published:** 2021-01-30

**Authors:** Frank Müller, Evelyn Kleinert, Nele Hillermann, Anne Simmenroth, Eva Hummers, Anna Zychlinsky Scharff, Christian Dopfer, Christine Happle, Alexandra Jablonka

**Affiliations:** 1 Department of General Practice, University Medical Centre Goettingen, Goettingen, Germany; 2 Department of General Practice, University Medical Centre Wuerzburg, Wuerzburg, Germany; 3 Department for Pediatrics, Hannover Medical School, Hannover, Germany; 4 Department of Paediatric Pneumology, Allergology, and Neonatology, Hannover Medical School, Hannover, Germany; 5 German Centre for Lung Research, Biomedical Research in End Stage and Obstructive Lung Disease/ BREATH, Hannover, Germany; 6 German Centre for Infection Research (DZIF), Partner Site Hannover-Brunswick, Braunschweig, Germany; 7 Department of Rheumatology and Immunology, Hannover Medical School, Hannover, Germany

## Abstract

**Background:**

Currently, health care systems worldwide are challenged with providing care to an increasing number of migrants, refugees, and displaced persons. In this article, we report on disease burden and drug prescription patterns in a large refugee cohort in Germany.

**Methods:**

We conducted a cross-sectional study of anonymized medical records including demographic data, diagnoses, and drug prescriptions in two refugee reception centres between 2015 and 2019. Refugees and migrants received medical assistance exclusively through the on-site clinics. Thus, this study represents all medical visits of the housed residents.

**Results:**

In total, n = 15531 diagnoses from n = 4858 patients in a cohort of n = 10431 accommodated refugees were recorded. N = 11898 medications were prescribed. Overall, 29.8% of all refugees sought medical attention. Half of the patients were female (49.6%), the average age was 23.8 years (SD [standard deviation] 17.0, min 0, max 81), and 41.5% were minors (<18 years). Most patients had Middle Eastern or Northern African origin (63.9%). The largest proportion of diagnoses belonged to the ICD (International Statistical Classification of Diseases and Related Health Problems) category “R” (miscellaneous, 33.5%), followed by diseases of the respiratory system (category “J”, 16.5%), or the musculoskeletal system (category “M”, 7.1%). Non-steroidal anti-inflammatory drugs were most frequently prescribed.

**Conclusions:**

This analysis in two large refugee centres in Germany shows that about one third of refugees seek medical attention upon initial arrival. Complaints are manifold, with a high prevalence of respiratory infections.

Due to the development of numerous humanitarian crises, primarily in the Middle East, Germany has hosted more than 2.3 million asylum seekers and refugees (AS&R) in the last decade [1]. Among western nations, Germany is host to one of the largest refugee populations [2]. Within the first fifteen months after entering Germany, asylum seekers have only restricted access to the health care system. During this time, an asylum seekers health care coverage is limited to emergency care (eg, in case of acute conditions or pain management for a chronic illness), acute dental care, and the provision of prescription drugs and basic medical appliances. Additionally, standard vaccinations are covered, as well as medical care during pregnancy and birth. All health services are free of charge. Furthermore, asylum seekers receive a mandatory medical assessment after claiming asylum.

Numerous studies show that refugee populations represent a particularly vulnerable patient group [3]. Many refugees have experienced psychological or physical trauma. This is reflected in increased rates of mental health issues such as posttraumatic stress disorders [4,5]. Many are affected by unsafe or exploitative working conditions and severe poverty in their countries of origin, during flight and in hosting nations [6]. A considerable number of refugees come from countries with destroyed or poorly developed health care systems [7]. Access to health services, adequate nutrition and sanitation is often not available to refugees during their flight [8].

Although hundreds of thousands of Asylum seeker and refugees (AS&R) have sought asylum in Germany in recent years, comparatively little is known about their medical needs and disease burden. Previous publications focused on communicable diseases such as HIV (human immunodeficiency viruses), tuberculosis, hepatitis, scabies, etc., as well as the vaccination status. [9-12]. Although the overall prevalence of HIV, hepatitis B and C and tuberculosis appear to be low, certain refugee subgroups are particularly affected by these diseases [9,13-15]. Thus far, few studies addressed the overall demand for medical care, or diagnoses and drug prescriptions in initial refugee reception settings [16-20]. To develop strategies for providing health care to migrant and refugee communities and enhancing service policies, we aim to describe patterns of diagnoses and drug prescription rates in this understudied population. To this end, we analysed medical encounters from two reception facilities in Germany, taking sociodemographic factors into account.

## METHODS

### Data collection and management

A retrospective analysis of all diagnoses (coded according to the International Statistical Classification of Diseases and Related Health Problems [ICD-10]) and prescriptions (coded according to Anatomical Therapeutic Chemical Classification System [ATC] and dosage form) of each consultation was performed. Information collected from health records included patients’ age, sex, refugee status, and nationality. Chart review was performed by medical students under the supervision of experienced medical doctors. All collected data was pseudonymised. In order to calculate prevalences over the course of the year, data on the number of accommodated residents for each day of the study were provided by the initial reception centres. Patients’ regions and countries of origin were recorded in the patient charts according to the official identity documents issued to the individual from the local government as part of the legal asylum process in Germany. For resettled refugees, where no country of origin could be determined, the last country of residence was coded instead. We grouped patients’ regions of origin in accordance with the World Bank Atlas of Sustainable Development Goals 2018 [21]. In cases of stateless patients or where the country of origin was unknown or under investigation, the origin of patients was coded as “stateless” and “unknown” respectively.

### Study population and setting

In this study, all medical encounters from two large initial reception centres in Germany were analysed. The first cohort (n = 1747 patients) was enrolled at an on-site medical unit in a reception centre for newly arriving AS&R in Celle, Northern Germany, between September 2015 and June 2016. This facility was a temporary shelter built to cope with the large influx of asylum seekers during the 2015 refugee crisis and closed when numbers of asylum seekers decreased in 2016. Parts of this cohort were previously described [22,23]. The second cohort (n = 3111 patients) was enrolled in a permanent reception facility in Friedland, Central Germany, from August 2017 to January 2019. A subset of this cohort has already been described [24]. Newly arrived asylum seekers were accommodated in these reception facilities for a transitional period, usually a few weeks to a few months, upon entering the country and claiming asylum. A second category of refugees called “resettlement refugees” were also accommodated in Friedland. These resettlement refugees have been recognized by the UNHCR (United Nations High Commissioner for Refugees) as particularly vulnerable, since they cannot return to their home country, nor can they stay in the country to which they have fled. As part of multilateral agreements, those refugees are offered permanently resettlement. Typically, these resettled migrants are selected in refugee camps in third party countries, mainly by UNHCR staff, and safely transferred to their country of destination in large groups.

**Figure 1:**
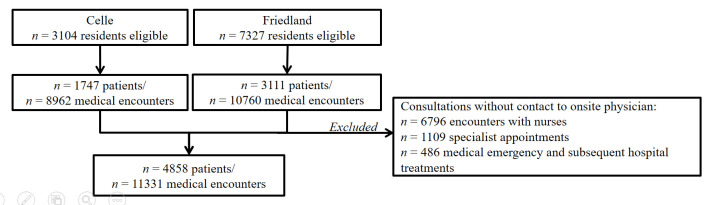
Flow diagram illustrating selection criteria of the study sample.

Both reception facilities were operated by the regional government of Lower Saxony and provided accommodation, meals, support from social services, and medical care. At both facilities, on-site medical units provided primary health care to all residents. Each on-site unit was staffed by nurses 24 hours per day, supplemented by on-site physicians during business hours on weekdays. AS&R could receive medical assistance exclusively through the on-site clinics; thus the study represents all medical visits by the housed residents. Any of the n = 10431 AS&R who were temporarily housed during the study period were eligible to participate in the study. For the analyses, we excluded all consultations without contact to an on-site physician (eg, medication dispensing by on-site nurses). A diagram illustrating selection criteria is shown in **Figure 1****.**

### Statistical analyses

Statistical analyses were performed using SPSS (Version 25, IBM, Armonk NY). Figures and maps were constructed using Google Spreadsheet (Alphabet Inc., Mountain View, CA). The distribution of characteristics in our sample was analysed (absolute and relative frequencies, means, standard deviations (SD)). We followed two different approaches: on the one hand, we examined the distribution of treatment cases and prescriptions as a whole and over the course of the year, and on the other hand, we characterized patient’s demographics and their prescriptions or diseases. Comparisons with regard to a certain characteristic always refer to two groups that share or do not share this characteristic, and not between the bearer of the characteristic and the distribution of this characteristic in the cohort as a whole. We used a χ2 test to test categorical variables and Mann-Whitney U test for testing metric and categorical variables. *P* values of equal or less than 0.05 were considered significant.

### Ethics compliance

This study was approved by the Research Ethics Board of the University Medical Centre Göttingen (Ethics approval No. 16/3/17) for the Friedland site and by the Institutional Review Board of Hannover Medical School (Ethics approval No. 3217-2016) for the Celle site. The study was carried out in compliance with guidelines on good clinical practice (GCP). We abstained from obtaining participants’ written informed consent as all data was collected by performing a retrospective chart review and processed in a pseudonymised manner. The decision to waive obtaining informed consent was approved by both the University Medical Centre Göttingen Ethics Comission and Hannover Medical School Ethics Commission for the entire study. Data collection for the Friedland site was conducted as part of the DICTUM-Friedland study, which was registered at the WHO clinical trial registry on September 29, 2017 (ID: DRKS00013076).

## RESULTS

**Figure 2:**
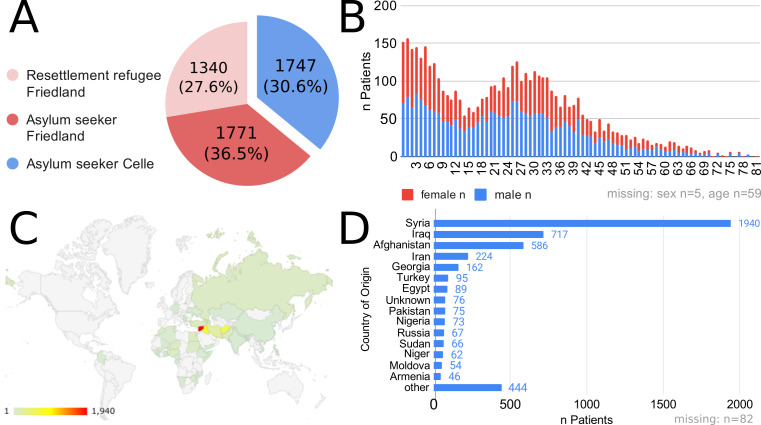
Cohort characteristics. **Panel A.** Distribution and refugee status of included patients in respective reception centres. **Panel B.** Age and sex distribution of all included subjects. **Panel C** and **Panel D.** regions and countries of origin of patients in the analysed cohort.

During the observational period, n = 10431 AS&R residents (n = 7327 in Friedland, n = 3104 in Celle, **Figure 2**, Panel A) lived in the two reception centres studied. Of all residents, 29.8% (n = 4858) sought medical attention with a total of n = 11331 consultations with on-site primary health care physicians. Residents that received medical care at least once are referred to in the following as “patients”.

Patients had an average of 2.33 consultations with doctors.

The mean age of patients was 23.8 years (SD 17.0, min 0, max 81). 41.5% (n = 2018) were children (<18 years old, thus legally minors in Germany). The sex ratio was nearly equal, with 49.6% female patients (**Figure 2**, Panel B). Most patients, n = 3050 (63.9%), came from the Middle East and North Africa (as defined by the World Bank Regional Groupings), followed by patients from South Asia (n = 672, 13.8%). The vast majority of patients came from Syria (n = 1940, 40.0%) and Iraq (n = 717, 14.8%). The largest proportion (72.4%, n = 3518) of patients were asylum seekers, while n = 1340 were resettled refugees. Patients’ country and regions of origin are shown in **Figure 2**, Panels C and DD.

Overall, we recorded n = 15531 diagnoses covering n = 814 different diagnostic codes (an average of n = 3.2 diagnoses per patient; Figure S1 in the **Online Supplementary Document**). In n = 785 consultations (6.9%), no diagnosis was coded. Frequencies and distribution of diagnoses are shown in **Table 1****.**

**Table 1:** Characteristics and frequencies of all patients’ diagnoses in according to ICD diagnostic groups

Patients' diagnoses (ICD-10 groups)	All Patients n = 4858	Prevalence in all residents	Female patients n = 2243			Age (years)	
**n (%)**	**(%)**	**n (%)**	**Proportion (%)**	***P*-value**	**mean** **±** **SD**	***P*-value**
A00-B99 − Certain infectious and parasitic diseases	624 (12.9)	6.0	328 (14.6)	52.6	0.001	17.9 ± 16.0	<0.001
C00-D49 −Neoplasms	30 (0.6)	0.3	18 (0.8)	60.0	0.129	38.4 ± 14.7	<0.001
D50-D89 − Diseases of the blood and blood-forming organs	37 (0.8)	0.4	27 (1.2)	73.0	0.001	27.0 ± 13.8	0.130
E00-E89 − Endocrine, nutritional and metabolic diseases	171 (3.5)	1.6	96 (4.3)	56.1	0.008	43.3 ± 18.4	<0.001
F01-F99 − Mental, Behavioral and Neurodevelopmental disorders	194 (4.0)	1.9	98 (4.4)	50.5	0.221	30.6 ± 13.9	<0.001
G00-G99 − Diseases of the nervous system	210 (4.3)	2.0	95 (4.2)	45.2	0.771	31.6 ± 14.4	<0.001
H00-H59 − Diseases of the eye and adnexa	300 (6.2)	2.9	129 (5.8)	43.0	0.248	26.2 ± 19.2	0.083
H60-H95 − Diseases of the ear and mastoid process	289 (6.0)	2.8	130 (5.8)	45.0	0.664	18.0 ± 15.6	<0.001
I00-I99 − Diseases of the circulatory system	230 (4.7)	2.2	115 (5.1)	50.0	0.239	47.3 ± 15.1	<0.001
J00-J99 − Diseases of the respiratory system	1735 (35.8)	16.6	795 (35.4)	45.8	0.679	18.5 ± 16.5	<0.001
K00-K95 − Diseases of the digestive system	710 (14.6)	6.8	313 (14)	44.1	0.217	28.2 ± 16.0	<0.001
L00-L99 − Diseases of the skin and subcutaneous tissue	595 (12.3)	5.7	332 (14.8)	55.8	<0.001	22.7 ± 16.9	0.046
M00-M99 − Diseases of the musculoskeletal system	719 (14.8)	6.9	307 (13.7)	42.7	0.040	34.6 ± 14.6	<0.001
N00-N99 − Diseases of the genitourinary system	227 (4.7)	2.2	177 (7.9)	78.0	<0.001	30.3 ± 12.4	<0.001
O00-O9A − Pregnancy, childbirth and the puerperium	44 (0.9)	0.4	44 (1.9)	100.0	<0.001	28.4 ± 7.1	0.014
P00-P96 − Certain conditions originating in the perinatal period	5 (0.1)	0.0	2 (0.1)	40.0	0.780	3.6 ± 4.5	0.003
Q00-Q99 − Congenital malformations, deformations and chromosomal abnormalities	28 (0.6)	0.3	17 (0.8)	60.7	0.123	16.9 ± 15.9	0.027
R00-R99 − Symptoms, signs and abnormal clinical and laboratory findings	2409 (49.6)	23.1	1166 (52)	48.4	0.002	22.3 ± 16.6	<0.001
S00-T88 − Injury, poisoning and certain other consequences of external causes	289 (6.0)	2.8	114 (5.1)	39.4	0.017	21.2 ± 15.6	0.009
Z00-Z99 − Factors influencing health status and contact with health services	490 (10.1)	4.7	330 (14.7)	67.3	<0.001	23.5 ± 13.5	0.666

ICD – International Classification of Diseases, SD – standard deviation

In total, n = 11898 drug prescriptions (n = 1.32 prescribed drugs per resident and month, n = 1.44 drugs per encounter) were given. In 30.9% of all consultations (n = 3502), no drug was prescribed. Frequencies and distribution of prescriptions are shown in **Table 2****.**

**Table 2:** Characteristics and frequencies of all prescribed medications by ATC grouping

	All patients n = 4858	Prevalence in all residents	Female patients n = 2243			Age (years)	
**Patients' prescribed medication (ATC groups)**	**n (%)**	**(%)**	**n (%)**	**Proportion (%)**	***P*-value**	**mean** **±** **SD**	***P*-value**
(A) Alimentary tract and metabolism	1110 (13.8)	10.6	528 (13.8)	47.6	0.836	31.0 ± 19.4	<0.001
(B) Blood and blood forming organs	173 (2.2)	1.7	94 (2.4)	54.3	0.085	38.7 ± 17.5	<0.001
(C) Cardiovascular system	326 (4.1)	3.1	164 (4.3)	50.3	0.366	50.2 ± 12.1	<0.001
(D) Dermatologicals	845 (10.5)	8.1	454 (11.8)	53.7	<0.001	21.5 ± 17.6	<0.001
(G) Genito-urinary system and sex hormones	121 (1.5)	1.2	113 (2.9)	93.4	<0.001	31.7 ± 13.3	<0.001
(H) Systemic hormonal preparations	73 (0.9)	0.7	51 (1.3)	69.9	<0.001	35.0 ± 17.1	<0.001
(J) Antiinfectives for systemic use	1136 (14.2)	10.9	563 (14.7)	49.6	0.214	22.2 ± 17.4	<0.001
(L) Antineoplastic and immunomodulating agents	28 (0.3)	0.3	5 (0.1)	17.9	0.001	41.7 ± 11.4	0.001
(M) Musculo-skeletal system	1426 (17.8)	13.7	604 (15.7)	42.4	<0.001	32.4 ± 14.6	<0.001
(N) Nervous system	1770 (22.1)	17.0	852 (22.2)	48.1	0.789	25.1 ± 18.5	0.026
(P) Antiparasitic products, insecticides and repellents	122 (1.5)	1.2	53 (1.4)	43.4	0.326	23.2 ± 14.8	0.182
(R) Respiratory system	2950 (36.8)	28.3	1338 (34.9)	45.4	0.001	17.0 ± 15.6	<0.001
(S) Sensory organs	297 (3.7)	2.9	138 (3.6)	46.5	0.625	23.1 ± 18.6	0.446
(V) Various	42 (0.5)	0.4	40 (1.0)	95.2	<0.001	25.6 ± 7.0	0.229

ATC – Anatomical Therapeutic Chemical Classification System, SD – standard deviation

### Frequent diagnoses

The most frequent categories were “symptoms, signs and abnormal clinical findings not elsewhere classified” (ICD-10 category “R”, n = 5201, 33.5% of all diagnoses). The most prevalent individual diagnoses occurring in our cohort were of the category “R”: “cough/R05” (n = 1124, 7.2% of all diagnoses), “pain of throat and chest/R07” (n = 854, 5.5% of all diagnoses), “fever/R50” (n = 734, 4.7% of all diagnoses), and “headache/R51” (n = 645, 4.2% of all diagnoses). Children and adolescents were more likely to present with “R” diagnosis (44.5% vs 38.7% in patients without any “R” group diagnosis, *P* < 0.001).

The second most common diagnosis group concerned the respiratory system (ICD-10 category “J”). The diagnosis of “acute nasopharyngitis [common cold]/J00” was the most frequent (n = 776, 5.0% of all diagnoses), followed by “acute upper respiratory infection/J06” (n = 561, 3.6% of all diagnoses), “acute bronchitis/J20” (n = 337, 2.2% of all diagnoses), and “acute tonsillitis/J03” (n = 289, 1.9% of all diagnoses). 56.8% of patients with a “J” diagnosis were children and adolescents (vs 33.1% in patients without any “J” diagnosis, *P* < 0.001).

The third most common diagnosis group was diseases of the musculoskeletal system and connective tissue (ICD-10 “M” group). Here, the most commonly coded diagnosis was “backpain/M54” (n = 387, 2.5% of all diagnoses), followed by “other joint disorder/M25” (n = 272, 1.8% of all diagnoses), “other soft tissue disorders/M79” (n = 264, 1.7% of all diagnoses), and “other disorders of muscle/M62” (n = 32, 0.2% of all diagnoses). Patients with “M” diagnoses were on average more than a decade older than patients who did not receive diagnoses in this category (mean age 34.6 vs 21.7 years, *PP<*0.001). Only a small proportion of children and adolescents received these diagnoses (11,7% vs 46.7% in patients without any “M” group diagnosis, *P* < 0.001).

The fourth most commonly used diagnosis group was that of diseases of the digestive system (ICD-10 “K” group). The most frequent diagnosis in this area was ”other disorders of teeth and supporting structures/K08” (n = 503, 3.2% of all diagnoses), followed by ”dental caries/K02” (n = 130, 0.8% of all diagnoses), ”other functional intestinal disorders/K59” (n = 110, 0.7% of all diagnoses), and ”gastritis and duodenitis/K29” (n = 109, 0.7% of all diagnoses). 27.6% (n = 196) of patients with a “K” diagnosis were children or adolescents (vs 43.9% in patients without a “K” diagnosis, *P* < 0.001).

The fifth most common diagnostic category was ailments concerning skin and subcutaneous tissue (ICD-10 “L” group). The most frequent used single diagnosis was “pruritus/L29” (n = 190, 1.2% of all diagnoses), followed by “other and unspecified dermatitis/L30” (n = 164, 1.1% of all diagnoses), “diaper dermatitis/L22” (n = 105, 0.7% of all diagnoses), and “acne/L70” (n = 60, 0.4% of all diagnoses). Children and adolescents were not significantly more affected by skin diseases than adults. (40.8% vs 41.6% children and adolescents in patients without any “L” group diagnosis, *P* = 0.685).

Among diagnoses that did not fall into the categories described above,”essential (primary) hypertension/I10” was common and diagnosed in n = 170 patients (3.5% of all patients). Patients receiving this diagnosis were significantly older than those without such a diagnosis (mean age 51.1 vs 22.8 years in patients without I10 diagnosis, *P* < 0.001). Also, “unspecified infectious gastroenteritis and colitis/A09” was prevalent within our cohort in n = 186 patients (3.8% of all patients). This diagnosis particularly affected children and adolescents (72.6% of cases), with a mean age of patients of 12.2 years (vs 24.3 years in patients without that diagnosis, *P* < 0.001). “Otalgia and effusion of ear/H92” were diagnosed in n = 147 patients (1.1% of all patients), and 58.5% of patients were children or adolescents (mean age 18.7 vs 24.0 years in patients without that diagnosis, *P* < 0.001). “Conjunctivitis/H10” was diagnosed in n = 133 patients (2.7% of all patients) consultations. No significant age differences between patients with conjunctivitis and those not diagnosed with this disease was detected (mean age 22.9 vs 23.8 years in patients without conjunctivitis diagnosis; *P* = 0.083). 4.0% of all patients received a diagnosis indicating a psychiatric disease or complaint (ICD-10 “F” group).

**Figure 3:**
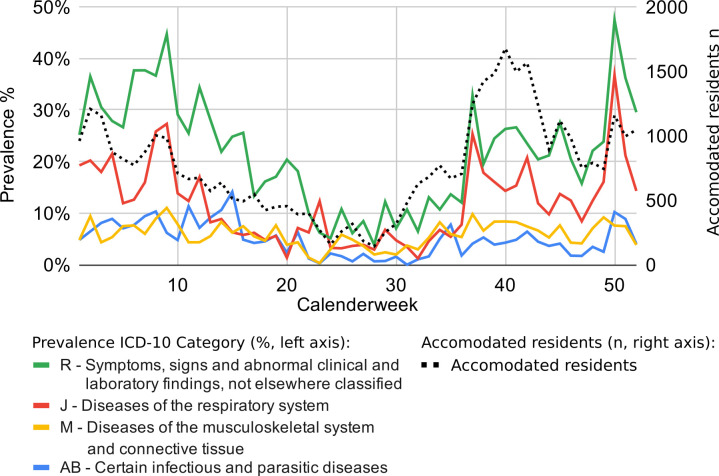
Prevalence of ICD codes per category (solid lines) and mean frequency of inhabitants in each week (striped black line) over the course of the year.

Disease prevalences displayed strong fluctuations over the course of the year (**Figure 3**). For example, diseases of the respiratory tract (“J”) were more common in autumn and winter. A total of 37% of the residents of the two centres sought medical help for such an illness at the end of December (50th calendar week). By the summer, the overall number of people demanding health care was considerably lower (eg, 1.6 encounters per inhabitant in June compared to 4.17 encounters per inhabitant in December). However, diagnoses of musculoskeletal diseases (“M”) and infectious diseases (“AB”) remained largely consistent throughout the year.

### Drug prescription

During the study period, n = 11898 medications were prescribed, comprising n = 467 distinct substances (Figure S2 in the **Online Supplementary Document**). The most commonly prescribed substance was ibuprofen (n = 2198, 18.5% of all prescriptions), followed by xylometazoline (n = 1060, 8.9% of all prescriptions), Hederae helicis folium (ivy leaf extract, which is often used as a herbal cough syrup for children in Germany [25], n = 945, 7.9% of all prescriptions), paracetamol/acetaminophen (n = 890, 7.5% of all prescriptions), amoxicillin (n = 603, 5.1% of all prescriptions) and metamizole (n = 476, 4.0% of all prescriptions). Almost half of the prescribed drugs were tablets or capsules (n = 5862, 49.3% of all prescriptions). Other common dosage forms were syrups or dry substances for mixing syrups (n = 2337, 19.6% of all prescriptions) as well as creams or ointments (n = 1214, 10.2% of all prescriptions).

Altogether, n = 3977 drugs of the ATC group “R” for respiratory ailments were prescribed (33.4% of all prescriptions). 36.8% of our patient cohort (n = 1932) received at least one drug prescription of this group. The most frequently prescribed substances of the “R” group were xylometazoline, ibuprofen (n = 1005, 8.4% of all prescriptions), Hederae helicis folium, and benzocain as throat lozenges (n = 353, 3.2% of all prescriptions). Children and adolescents more commonly received medication than adults (58.5% vs 30.3% in patients without any “R” prescription, *P* < 0.001).

In total, n = 1904 prescriptions of the ATC “N” group were given, comprising drugs with an effect on the nervous system. These medications contributed 16.0% of all prescribed drugs. 22.1% of all patients in our cohort received at least one drug of the “N” category. By far the most prescribed medications in this group were paracetamol/acetaminophen (n = 890, 7.5% of all prescriptions) and metamizole (n = 476, 4.0% of all prescriptions), followed by mirtazapine (n = 93, 0.8% of all prescriptions) and quetiapine (n = 37, 0.3% of all prescriptions). Children and adolescents represented 40.8% of the patients receiving prescriptions of the “N” category (vs 41.8% in patients without any “N” group prescription, *P* = 0.557).

A total of n = 1487 prescriptions of the ATC “M” group treating complaints of the musculoskeletal system occurred, representing 12.5% of all prescribed drugs. 17.8% of all patients in our cohort received “M” group medications. The most prescribed substance of this group was ibuprofen (n = 1185, 10.0% of all prescriptions) followed by topical diclofenac (n = 161, 1.4% of all prescriptions), oral diclofenac (n = 113, 0.9% of all prescriptions), and topical ibuprofen (n = 7, 0.1% of all prescriptions). Only 16.9% of these patients were children and adolescents (vs 48.3% in patients without any “M” group prescription, *P* < 0.001).

A total of n = 1161 prescriptions were drugs of the ATC “J” group (anti-infective agents for systemic use), which accounted to 9.8% of all prescriptions. 14.2% of all patients in our cohort received at least one of these drugs. The most frequently prescribed medications were amoxicillin (n = 603, 5.1% of all prescriptions), phenoxymethylpenicillin (n = 76, 0.6% of all prescriptions), cefuroxime (n = 76, 0.6% of all prescriptions), and ciprofloxacin (n = 55, 0.5% of all prescriptions). 45.4% of patients receiving these prescriptions were children and adolescents (vs 40.6% in patients without any “J” group prescription, *P* = 0.007).

Other frequently prescribed drugs not mentioned above were proton pump inhibitors such as pantoprazole (n = 270, 2.3% of all prescriptions) and omeprazole (n = 150, 1.3% of all prescriptions) as well as the antihistamine diphenhydramine (n = 136, 1.1% of all prescriptions).

**Figure 4:**
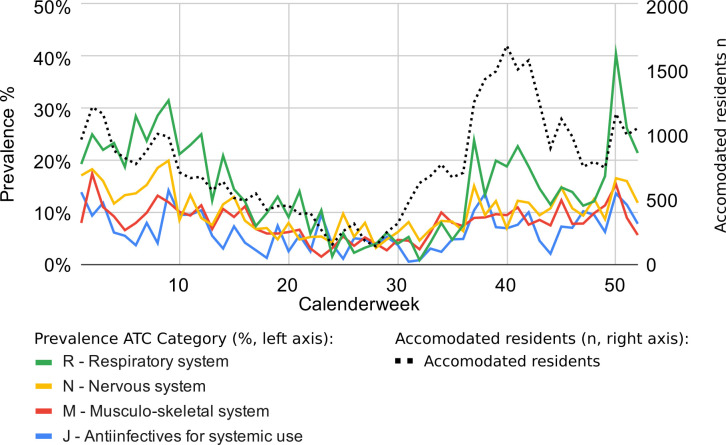
Prevalence of ATC grouped prescriptions (solid lines) and mean frequency of residents in each week (striped black line) over the course of the year.

Prescription prevalence over the course of the year correlated with the increase of infectious disease diagnoses, with higher numbers in fall and winter (**Figure 4**). For example, drugs from the ATC Group “R”/Respiratory system were mainly prescribed during the colder seasons. In the 50th calendar week, 40% of all residents of the reception centre were prescribed a drug from this group. Such periodicity was also observed for other drug categories, although not to the same extent.

## DISCUSSION

We present comprehensive data on diagnoses and prescribed medication in a large and current cohort of newly arriving AS&R entering Germany, showing a wide variety of diseases and drug prescription in this population. The average age in the analysed cohort was 23.8 years with almost half of the AS&R being minors, and most came from the Middle East or Northern Africa. These demographics are roughly in line with current immigration statistics in Germany and Europe [1,26].

There has been extensive migration towards Western Europe in recent years, especially from the Middle East and Africa, due to war, political conflict and economic factors. Primary health care and basic medical services are essential components in responding to this crisis [27]. The UNHCR estimates that about 25 million people are fleeing worldwide, and Germany has hosted and accepted many AS&R during the current crisis [28]. As such, the German health care system has been particularly challenged to provide adequate and high quality medical care to all refugees [29].

Still, uncertainties about reasons for and frequency of health care utilisation by AS&R remain [30,31]. To address this issue, we combined data from two large cohorts of AS&R living in reception centres in northern and central Germany and analysed data on symptoms and diagnoses of n = 4858 patients in a cohort of more than 10.000 asylum seekers and refugees accommodated in these facilities. 29.8% of all AS&R sought on-site medical attention at least once, in addition to the mandatory health check-up required by asylum law (§ 62 German Federal Asylum Law). As previously described for a part of this cohort, utilisation is highest in the first week after arrival and decreases significantly afterwards [32]. Refugees sought help due to different complaints, but most diagnoses and medication prescriptions fell within the domain of general practice. As such, our data suggests that most consultations for AS&R can be managed by general practitioners and dentists.

The restrictions on the provision of medical care to refugees enacted by German legislation attempt to treat exclusively unavoidable and acute diseases. This practice is viewed critically by medical professionals [33] and is regarded as ineffective and cost-intensive [34]. These regulations also prevent effective socio-medical interventions which could promote self-efficacy in people who have fled, both in terms of managing their situation in an unfamiliar host country and coping with illness. This could reduce the use of health services for minor complaints.

While interpreting these results, it must be taken into account that AS&R were accommodated for only a few months. Our results therefore only reflect the provision of health care to refugees in a short period directly after their arrival in Germany. It has been shown that health care utilisation by AS&R is strongly influenced by the ease of access to medical services [35]. As residents of the facility had on-site general practitioners readily available, and all medical appointments and medication were free of charge, barriers to care were low. This may explain the high rate of health care utilisation compared to other studies [36,37]. Additionally, it should be considered that, in contrast to other countries, specially trained nurse practitioners who provide independently consultations are not common in the health care system in Germany. Physicians are therefore often entrusted with the treatment of minor illnesses that would typically be treated by nurse practitioners in other countries which may have increased the number of consultations.

On average, 3.2 diagnoses were coded for each patient. Especially frequent were diagnoses of the “R” group, which were coded in 49.6% of all patients. The “R” group is a widely used ICD-10 category in primary care, as it allows the coding of unspecific symptoms rather than clear diagnoses [38]. This may be due to the fact that fixed diagnoses are often not possible and less relevant for treatment as compared to “working diagnoses” based on symptoms. However, the use of “R” group diagnoses might be even more common in the setting of providing health care to AS&R, where definite diagnoses are more difficult to determine due to first-time consultations as well as language and cultural barriers.

As expected, infectious diagnoses and symptoms thereof such as “cough”, “throat pain” and “fever” were highly prevalent in our analyses, and the category “respiratory infections/J” was the second most common in our patient cohort. In our analysis, 35.8% of all patients received at least one diagnosis within this category and 36.8% of the analysed patients received at least one drug prescription from the “respiratory system/R” group. The prevalence of respiratory illnesses showed a classic seasonality with higher rates in autumn and winter. In addition, drugs from the “J” group (systemic anti-infective agents) were prescribed to 14.2% of all patients. Concerning the high rate of viral pathogens causing respiratory symptoms in our cohort, this number seems high and should be examined in further investigations. This is in line with findings from Israel, Switzerland and Italy, where respiratory infections and signs and symptoms thereof were amongst the most common reason for presentation [39-41]. In contrast, data from German general practitioners' practices show that far more diagnoses are given for chronic diseases: the German Federal Health Report lists hypertension as the most frequent diagnosis (38.7% of all treatment cases) followed by dyslipoproteinemia (26.4% of all treatment cases), but also back pain (16.7% of all treatment cases) [42].

We previously described the increased demand for medical care in reception centres during fall and winter [23,43]. Communicable diseases, particularly respiratory infections, have been previously described to be very common in AS&R [16,44,45]. This may in part be due to the young average age of this population, as almost half of our cohort were minors and acute respiratory infections are among the most frequent diagnoses in German-born children and adolescents [46]. However, this finding is also of particular concern in the context of the current COVID-19 pandemic [47]. This unprecedented health emergency represents a significant threat in particular to migrants and refugees who - due to malnutrition, crowded housing, poor sanitation, and other factors – are at increased risk of contracting SARS-CoV-2 (Severe acute respiratory syndrome coronavirus 2) and suffer from severe disease courses [48].

The considerable seasonal fluctuations in health care utilisation and especially the prevalence of respiratory diseases indicate how health care services can further adapt to the needs of this population.

We also observed a noticeably high prescription rate of NSAIDs (Nonsteroidal anti-inflammatory drug), predominantly over-the-counter (OTC) drugs such as ibuprofen and paracetamol (acetaminophen), but also the prescription-only drug metamizole. These were predominantly prescribed for respiratory symptoms, back pain, and headaches. In this context, even though the majority of prescribed drugs were OTC, the considerable range of potential side effects should be emphasized [49]. The high prescription rate of NSAID may also be based on the fact that communication on non-medication treatment regimens, eg, advice on physical activity for patients with lower back pain or watch-and-wait approaches are harder to communicate in intercultural settings [50]. Refugees may also prefer to access and store analgesics drugs at home for quick access, as described by Bruna Tresànchez-Lacorte et al. [51].

Previous reports have established that refugee populations are at an increased risk for psychological and psychiatric issues [52,53]. Complaints from this diagnostic group represented 4.0% of patients in our analysis which seems low in comparison to the existing evidence on a higher burden of mental health in this population and in comparison with a German non-refugee cohort, were a comparable number of patients with psychological issues (3.3%) was reported [54].

Certain limitations should be taken into account when interpreting our data. Due to the dynamic nature of the refugee crisis and changing compositions of the refugee population in Germany, the results from our cohort, while large and representative of current demographics, should not be extrapolated too far, eg, for other refugee populations. In addition, diagnoses for this cohort were ICD-classified post-hoc, possibly introducing a categorization bias. Since our study refers to the diagnoses and prescriptions made in the context of health care utilisation, they characterize patients’ demands, needs, and reasons for treatments and therefore investigates subjective disease burden.

Nevertheless, our findings can be used as a resource to better adapt health care provision to refugees as it provides comprehensive information on distribution of health complaints and drug therapy in a large, representative, and current cohort with an observation period of 27 months. Taken together, our analysis shows that about one third of AS&R seek on-site medical attention upon initial reception and that complaints are manifold and can, to large extent, be treated by general practitioners and dentists. Despite existing prejudices, our results do not show that refugees suffer extensively from infectious, dangerous or exotic diseases or put a high financial burden on the social system.

Providing primary care to refugees is challenging, but constitutes an essential humanitarian service, especially in the context of the current global pandemic. We hope the data presented here may aid in designing adapted health care strategies for the particularly vulnerable group of refugees and asylum seekers.

## Additional material

Online Supplementary Document
