# Supplementary material for: Disease burden in a large cohort of asylum seekers and refugees in Germany
Source: J Glob Health. 2021 Jan 30;11:04002. doi: 10.7189/jogh.11.04002 (PMC7897448; doi:10.7189/jogh.11.04002)
Supplement: Online Supplementary Document [file jogh-11-04002-s001.pdf]

Figure S1.

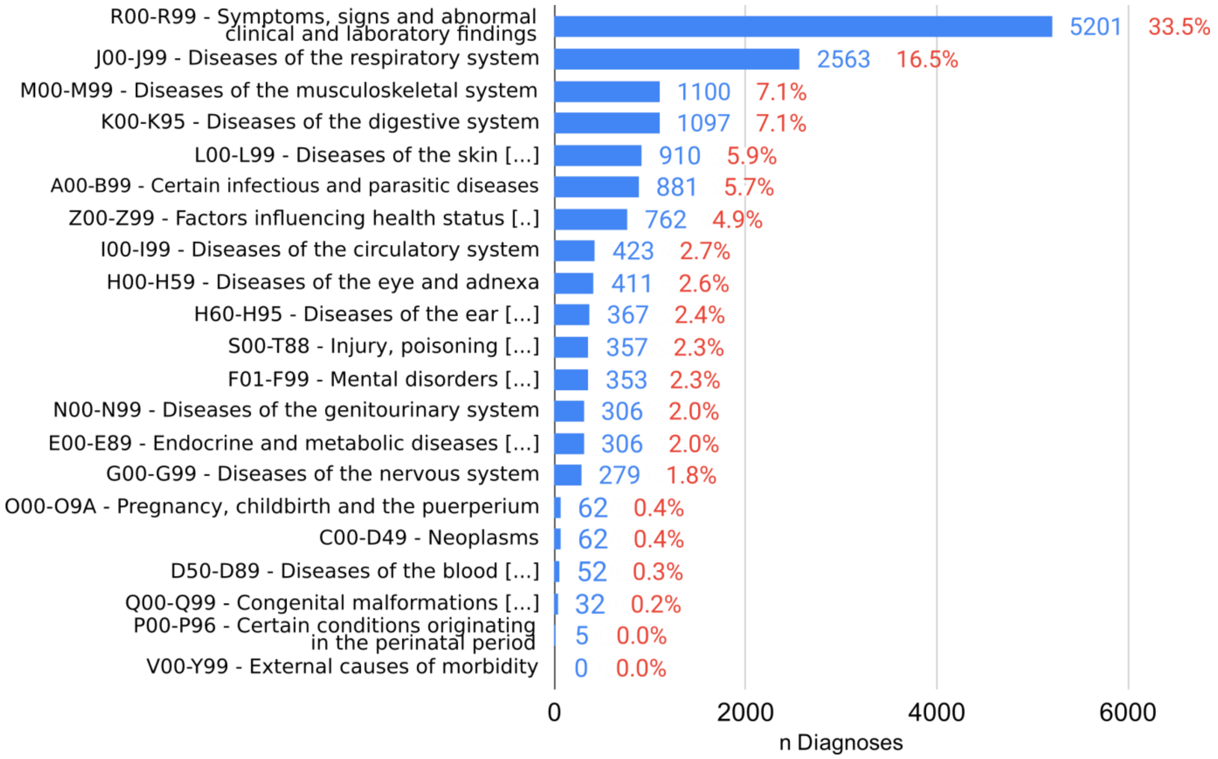

Figure S1: Proportion of diagnoses categorised in ICD main groups in relation to all recorded diagnoses (n=15531).

Figure S2.

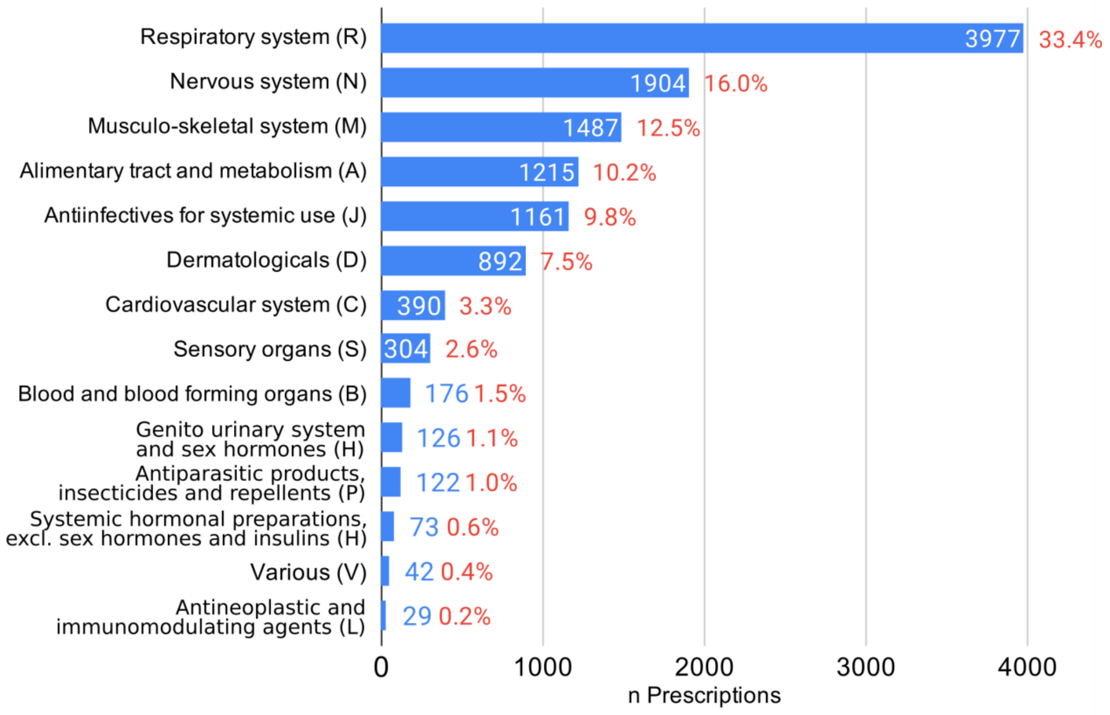

Figure S2: Proportion of prescribed drugs according to ATC groups in relation to all recorded prescriptions (n=11898).
